# Preparation, Thermal Analysis, and Mechanical Properties of Basalt Fiber/Epoxy Composites

**DOI:** 10.3390/polym12081785

**Published:** 2020-08-10

**Authors:** Konstantinos Karvanis, Soňa Rusnáková, Ondřej Krejčí, Milan Žaludek

**Affiliations:** 1 Department of Production Engineering, Faculty of Technology, Tomas Bata University in Zlín, Vavrečkova 275, 760 01 Zlín, Czech Republic; rusnakova@utb.cz (S.R.); zaludek@utb.cz (M.Ž.); 2 Department of Polymer Engineering, Faculty of Technology, Tomas Bata University in Zlin, Vavrečkova 275, 760 01 Zlín, Czech Republic; okrejci@utb.cz

**Keywords:** basalt fiber, epoxy composite, glass transition temperature, DMA, TMA, creep recovery, stress-relaxation

## Abstract

In this study, basalt fiber-reinforced polymer (BFRP) composites with epoxy matrix, 20 layers, and volume fraction of fibers *V_f_* = 53.66%, were prepared by a hand lay-up compression molding combined method. The fabric of the basalt fibers is in twill 2/2 weave. Through dynamic mechanical analysis (DMA), their viscoelastic behavior at elevated temperatures and in various frequencies was explored, whereas thermomechanical analysis (TMA) took part in terms of creep recovery and stress-relaxation tests. Moreover, the glass transition temperature (*T_g_*) of the BFRP composites was determined through the peak of the tanδ curves while the decomposition of the BFRP composites and basalt fibers, in air or nitrogen atmosphere, was explored through thermogravimetric analysis (TGA). The mechanical behavior of the BFRP composites was investigated by tensile and three-point bending experiments. The results showed that as the frequency is raised, the BFRP composites can achieve slightly higher *T_g_* while, under the same circumstances, the storage modulus curve obtains a less steep decrease in the middle transition region. Moreover, the hand lay-up compression molding hybrid technique can be characterized as efficient for the preparation of polymer matrix composites with a relatively high *V_f_* of over 50%. Remarkably, through the TGA experiments, the excellent thermal resistance of the basalt fibers, in the temperature range 30–900 °C, was revealed.

## 1. Introduction

Nowadays, fiber-reinforced polymer (FRP) composites are broadly used, with the most use in critical applications. These composites have proved their significant mechanical behavior in various applications whereas research on them, using various materials and methods for their production, is still ongoing. In particular, FRP composites consist of a polymer matrix reinforced with fibers. The usual applications of the FRPs are in the aerospace, marine, automotive, and construction industries [1]. Additionally, due to their high strength-to-weight ratio, high stiffness-to-weight ratio, corrosion resistance, and light weight, the FRP composites are appealing in civil engineering applications [2]. It must be noted that these materials have generally remarkable costs, so the applications must verify them and the parameters such as fibers’ architecture and composites’ production method affect the properties of the FRP composites to a great extent.

The most commonly used fibers in the FRP composites are the carbon, glass, aramid, and basalt; boron and silicon carbide fibers are also used, but in limited amounts. When long fibers are applied as reinforcement phase, the composite obtains characteristics familiar for structural applications because of the remarkable ability of these fibers to carry loads.

A promising material for usage in various applications is the basalt, which is solidified volcano lava. The basalt fibers are attractive to be used as reinforcement phase in composites because they combine superior properties with low price. In particular, basalt fibers can be fabricated through the use of basalt rocks [3]. When these fibers are embedded in a polymer matrix, the named basalt fiber-reinforced polymer (BFRP) composites are formed. The stiff and brittle nature of the basalt fibers is associated with their disadvantages [4].

During the production of the basalt fibers, shattered basalt rocks are melted at 1400 °C and the molten material is drawn [5]. The continuous basalt fibers are fabricated with technology similar to the E-glass, with the primary discrepancy that the latter are produced through a complex group of materials, whereas the basalt filament is produced through the melting of basalt rocks without other additives [6]. Corresponding to the degree of the contained SiO_2_, the basalt materials are categorized to alkaline basalts (up to 42% SiO_2_), midly acidic basalts (43% to 46% SiO_2_), and acidic basalts (more than 46% SiO_2_) [7].

One of the most common polymers, which is used as a matrix in the FRP composites, is epoxy. Epoxy is primarily used for aerospace composites, but its long curing time makes it not the first choice in automotive applications where polyester, vinyl ester, or polyurethane polymer matrices are preferred due to their lower curing time than the epoxy [8].

The FRP composites production methods are mainly divided to autoclave and out of autoclave (OOA) methods. The former include the use of autoclave, with which high quality FRP composites are produced, but with significant costs for operation and energy, whereas the latter methods are more economic and not so high level equipment needed. The lay-up techniques are those in which layers are stacked, and the most familiar technique of them is the hand lay-up. In this method, the laminate, usually with fiber fabric as reinforcement phase, is prepared by placing ply over ply by hand until the desired thickness is achieved by the use of a roller as the resin is applied on the laminate and as the excessive amount of it is removed. The hand lay-up technique has two main disadvantages; it is difficult to achieve uniform distribution of the matrix, resulting in non-uniform percentage of fibers/matrix in the composite laminate, and that during the curing, usually there is no pressure on the laminate, which in turn increases the porosity of the composite plate. In this research, after the preparation of the lay-up of the BFRP composite plate and during its curing, compression molding was used to eliminate its porosity.

One of the main disadvantages of the FRP composites is their low thermal resistance and, generally, their weakness at high temperatures, something which is mainly caused by the low thermal resistance of the polymer matrix. The thermal behavior of the FRP composites is investigated through thermal analysis, which is the study of the materials’ properties in accordance with the temperature; measurement of properties under a specific temperature range or in an isothermal situation. Familiar experiments of this sector are the dynamic mechanical analysis (DMA), thermomechanical analysis (TMA), and thermogravimetric analysis (TGA). Generally, the materials’ behaviors are influenced by high temperatures, so it is necessary for these to be tested and verified under these circumstances.

DMA is used for the investigation of the rheology of the materials. By this technique, materials’ properties such as storage modulus, loss modulus, tanδ, and glass transition temperature (*T_g_*), under various temperatures, frequencies, forces, and deformation modes can be determined. Moreover, by DMA, the properties of viscoelastic materials, like the polymers, can be explored. With the term viscoelastic, materials which exhibit both elastic and viscous characteristics, when they are deformed, are described. Additionally, another characteristic of the DMA experiments is that the deformation can be applied on the specimens through various ways, depending on the instrument, such as three-point bending, single cantilever and dual cantilever bending, tension, compression, and shear mode. It must be noted that, in DMA tests, it is important the measurements to take part in the linear viscoelastic region of the materials.

Furthermore, through the DMA, the glass transition temperature (*T_g_*) can be specified. The *T_g_* is an important point in the mechanical behavior of the materials and usually, at this temperature, the polymers change phase from a solid state to a rubbery. However, *T_g_* is not very accurate defined whereas there are many methods for its determination such as peak of loss modulus or tanδ curves, so it is essential the method which was followed for its determination to be described. Furthermore, the *T_g_* is quite different from the melting temperature (*T*_m_), because the melting point is the beginning of the materials’ melting whereas at *T_g_* the materials becomes softer [9].

During the real life applications of the materials, forces are exerted on them for long periods of time so it is essential their properties to be studied in dependence with the time. Through stress-relaxation and creep recovery tests the materials’ time dependent behavior can be investigated [10]. It should be noted that, because the polymer matrix has viscoelastic behavior, polymer matrix composites show viscoelastic behavior with the stress and strain to be dependent on time and temperature [11]. In particular, the long molecular chains of the polymer matrix cause the viscoelastic phenomenon of the polymer composites [11]. Contrastively with the solid materials, the viscoelastic, when they are under a constant load, do not exhibit a constant deformation but they continue to flow with the time—the phenomenon named “creep” [12]. Additionally, the viscoelastic materials have time-dependent behavior and permanent deformation [13].

During a creep experiment, constant stress is applied on the material at isothermal temperature, and the arising strain is recorded in accordance with the time [14]. The parameters which affect the creep behavior of FRP composites are temperature, frequency, applied stress, and the interface between polymer-fibers. In the stress-relaxation experiment, a constant extension is applied on the sample and the reducing force is recorded as the time is increased.

Various properties of BFRP composites have been investigated by researchers. Huaian Zhang et al. [15] studied the effects of strain rate and temperature on the tensile properties of BFRP composites which were produced through vacuum-assisted resin infusion technique. 

Using two different matrices, vinylester resin and epoxy resin, and the same type of basalt fibers as reinforcement, C. Colombo et al. [16] produced two types of basalt fiber-reinforced composites, and they explored them experimentally through static tensile tests, static compression tests, static delamination tests, fatigue tests, and stepwise tests. The results showed that the basalt fiber-reinforced composites with epoxy matrix had higher ultimate tensile strength and ultimate compressive strength than the one with vinylester matrix [16]. 

T. Bhat et al. [17] studied the fire structural properties of a basalt fiber-reinforced polymer laminate under compressive loading and compared them with the properties of an E-glass fiber composite which had the same fiber content, ply orientation, and polymer matrix. Dynamic mechanical analysis was performed on BFRP plates by Zhongyu Lu et al. [18].

V. Lopresto et al. [19] fabricated, through vacuum bag technology, E-glass and basalt fiber-reinforced plastic laminates, and they investigated them in terms of tensile, bending, shear, compression, and impact tests. A study about the tensile, shear, and impact strengths of basalt fiber-reinforced unsaturated polyester composites with and without acid and alkali treatments of the fabrics was conducted by V. Manikandan et al. [20].

The influence of elevated temperatures on the flexural fatigue behavior of a pultruded basalt fiber-reinforced epoxy plate was studied by Zike Wang et al. [21]. In particular, the BFRP specimens were subjected to elevated temperature treatment in an oven at 150 °C and 250 °C for 0.5, 1, and 2 h [21].

Farzin Azimpour Shishevan et al. [22] investigated the low-velocity impact behavior of BFRP composites. In particular, in this study, BFRP and carbon fiber-reinforced polymer (CFRP) composites were produced through vacuum-assisted resin transfer molding (VARTM) method and they were tested to low-velocity impact experiments with different energy magnitudes [22].

Salvatore Carmisciano et al. [23] produced basalt and E-glass woven fabric reinforced composites. Particularly, these composite laminates were fabricated by a resin transfer molding (RTM) system, and through various tests, it was revealed that the basalt fiber composites exhibited higher flexural modulus and interlaminar shear strength but lower flexural strength compared to the composites containing E-glass fibers [23].

P. Amuthakkannan and V. Manikandan [24] studied the free vibration and dynamic mechanical properties of surface modified basalt fiber-reinforced polymer composites. In particular, by hand lay-up method, they fabricated untreated, acid-treated, and base-treated basalt fiber composite and investigated them through DMA [24].

However, a research study of BFRP composites, which have volume fraction of fibers (*V_f_*) over 50%, representing enough experimental results, both for the mechanical and the thermal behavior of these compounds, appears to be missing. At this point, it must be noted that in the FRP composites’ industry, the introduction of natural fibers, in percentage of 50% and over in a composite, while maintaining good mechanical behavior of it, is of great interest as much from an ecological point of view as from mechanical investigation aspects. In order to gain a better understanding of an FRP composite, due to its viscoelastic matrix, several thermal analysis techniques must be employed to study and characterize it.

In the present study, BFRP composites, consisting of 46.4% epoxy and 53.6% of basalt fibers, volume fraction percentages determined through TGA, in 20 layers, were successfully prepared by a hand lay-up compression molding combined technique and their dynamic mechanical properties, in terms of storage modulus, loss modulus, tanδ, and glass transition temperature were determined. Through the maximum values of the tanδ curves, the glass transition temperatures of them were defined while TMA was performed in the modes of creep recovery and stress-relaxation experiments. Moreover, TGA was used for the exploration of the decomposition of basalt fibers and BFRP composites, as well as for the determination of the weight fraction of reinforcement phase and matrix, whereas the mechanical behavior of the BFRP composites was investigated through tension and three-point bending experiments.

## 2. Experimental

### 2.1. Materials

**Figure 1:**
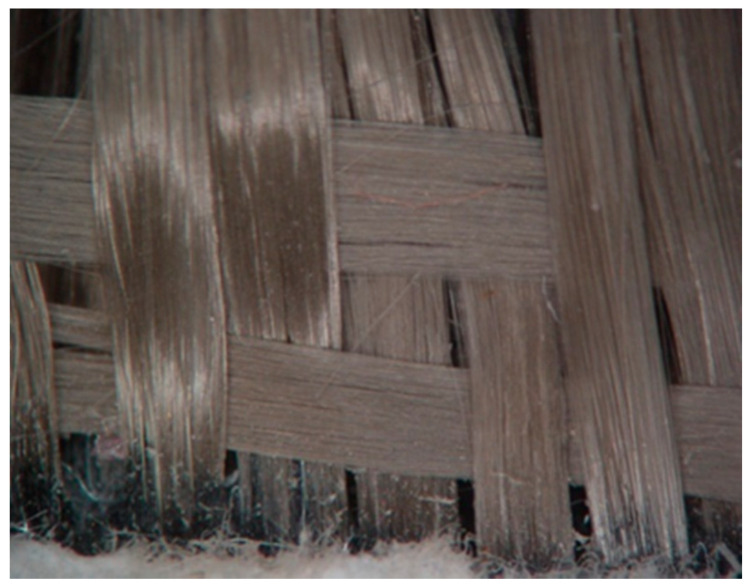
The used basalt fibers. The photo was taken with the Carl Zeiss Stemi 2000C Microscope (Jena, Germany) from the edges of the mold.

For the preparation of the BFRP composites, a mixture of Epoxy resin Epidian^®^ 652 CIECH Sarzyna S.A (Cieszyn, Poland) and hardener TFF CIECH Sarzyna S.A (Cieszyn, Poland), in a mixing ratio of 100:27 parts per weight, was used as matrix. A fabric of basalt fibers 235 g/m^2^, in twill 2/2 weave, supplied by Havel Composites (Cieszyn, Poland) was used as reinforcement phase (Figure 1). The fact that the specific weight of the used basalt fibers is 2.67 g/cm^3^ must be stressed. For the production of the BFRP composites, the steps described below were followed. By hand lay-up technique, a laminate composed of 20 layers of polymer fibers was prepared, and then it was placed between two rectangular metal plates forming a mold. It must be noted that the polymer matrix was applied on the fibers fabric through a roller. Consequently, this system was placed in a compression machine, where it was pressed under 20 MPa for 24 h, at a laboratory temperature of 24 °C. Then, the composite plate was left for curing at room temperature for a week, and finally, specimens were cut in the desired dimensions through water jet and mechanical cutting.

### 2.2. General Experimental Conditions

In all of the experiments, both of thermal analysis and mechanical behavior, the specimens were in storage in a laboratory environment at 24 °C for not less than 40 h prior of the tests. During the experiments, the conditions in the laboratory were at a temperature of 23 °C and humidity of approximately 50%. 

### 2.3. Dynamic Mechanical Analysis (DMA)

**Figure 2:**
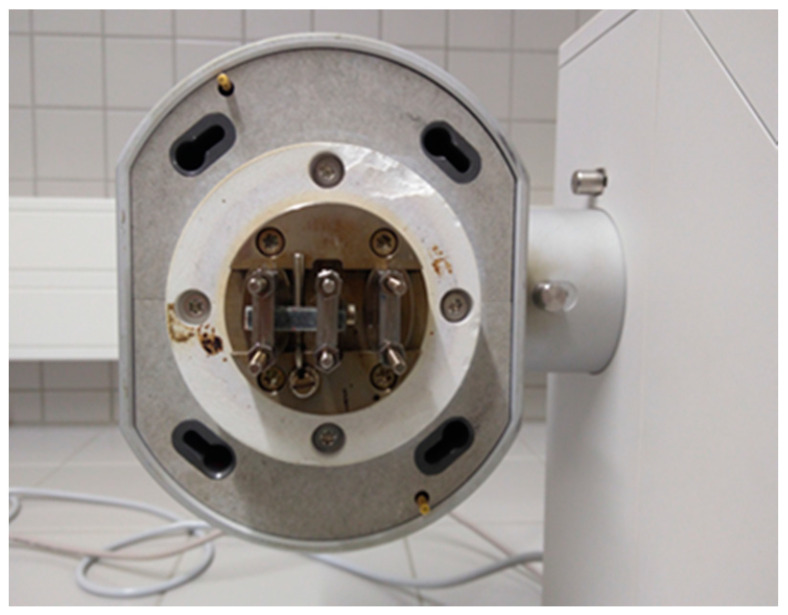
DMA specimen on the single cantilever mode.

The DMA experiments were performed by the DMA 1 instrument, from METTLER TOLEDO (Schwerzenbach, Switzerland), with using the STARe Software and under single cantilever configuration (Figure 2). The dimensions of the rectangular shape specimens were 25 mm × 5.7 mm × 2.1 mm (length × width × thickness).

Firstly, for the determination of the linear viscoelastic region of the BFRP composites, strain sweep tests over the range 1–31 μm were performed at 25 °C and 1 Hz frequency.

Next, temperature sweep tests were conducted over the temperature range of 30–180 °C, with a heating rate of 2 K/min under three different frequencies: 1, 5, and 10 Hz. The displacement amplitude was adjusted to be 8 μm. Moreover, the *T_g_* temperatures of the BFRP composites was determined through the peak of the tanδ and they are presented in Table 1.

### 2.4. Thermomechanical Analysis (TMA)

The creep recovery and stress-relaxation tests were carried out by the METTLER TOLEDO DMA 1 (Schwerzenbach, Switzerland) instrument, with the STARe Software and under TMA mode and three-point bending configuration. For both types of the experiments, creep recovery and stress-relaxation, the specimens were in rectangular shape with dimensions 40 mm × 5.7 mm × 2.1 mm (length × width × thickness) with the span length between the supports to be 30 mm. Specifically, the particular specifications of these tests are described below.

#### 2.4.1. Creep Recovery Tests

In the first type of the creep recovery experiments, at isothermal 25 °C, a force of 1, 3, or 5 Newton was applied on the specimens for 30 min and then the recovery behavior of them was recorded under 0 N for 120 min.

The second type of the creep recovery tests took part under three different temperatures. In detail, 1 N was applied on the specimens whereas the temperature was 25, 50, or 75 °C and the recovery of them, under 0 N, was measured for 120 min.

#### 2.4.2. Stress-Relaxation Tests

The stress-relaxation tests were performed at three different temperatures: 25, 50, and 60 °C. In detail, during these experiments, 20 μm extension was applied on the specimens and their time-dependent stress was being measured for 60 min. It should be noted that experiments were performed at 75 °C, but they were failed due to the small resistance of the BFRP sample in deformation at this temperature; the force was always falling in negative values.

### 2.5. Thermogravimetric Analysis (TGA)

The TGA was performed with the TGA Q50 Thermogravimetric Analyzer, from the TA Instruments (New Castle, DE, USA), in the temperature range 30–900 °C, with heating rate 10 °C/min, in air or nitrogen atmosphere for two different separate runs. It must be noted that the TGA tests were setting and performed with the Thermal Advantage Release 5.4.0 software and the results were evaluated with the TA Instruments Universal Analysis 2000 version 4.5A program. In particular, during these experiments, samples with weights of approximately 60 mg were placed in an alumina crucible, and the total flow was set at 100 mL/min; balance purge flow at 40 mL/min, and sample purge flow 60 mL/min. It must be noted that the complex structure of the BFRP composites requires enough mass during the TGA experiments, so as to be excluded reliable conclusions for its decomposition and that is why the weight of the TGA samples was relative big. 

### 2.6. Mechanical Properties

The flexural strength of the BFRP composites was determined at room temperature using the testing machine Zwick/Roell 1456 (Ulm, Germany) with the software testXpert^®^ II V2.1. In particular, the three-point bending experiments were exhibited with specimens of dimensions 75 mm × 10 mm × 2.1 mm (length × width × thickness) with the span length-to-depth ratio of 20:1, whereas the crosshead speed was set to 1 mm/min. For reliability in the measurements, four specimens were tested, and the average values of the flexural characteristics are presented in Table 2.

**Figure 3:**
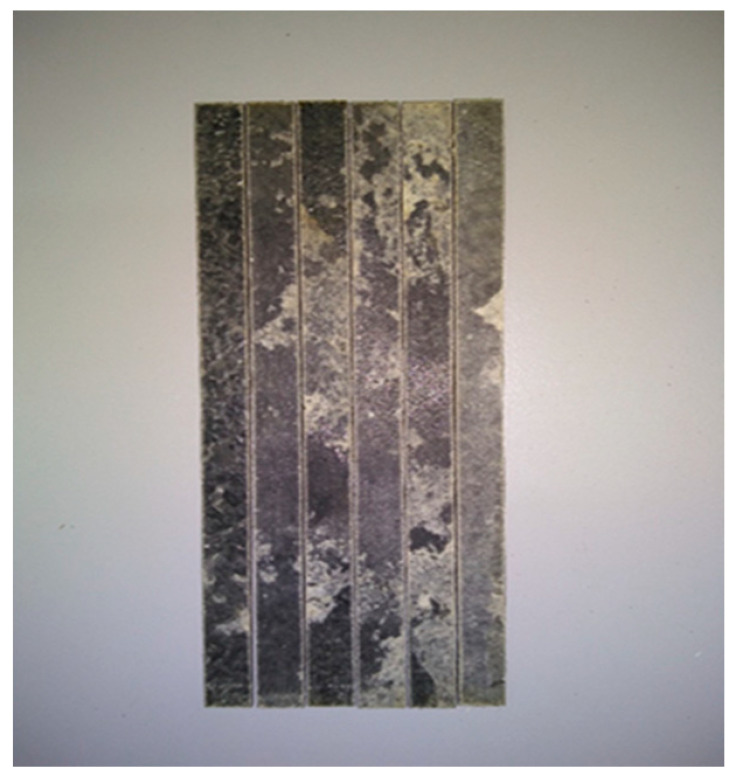
Tension samples.

**Figure 4:**
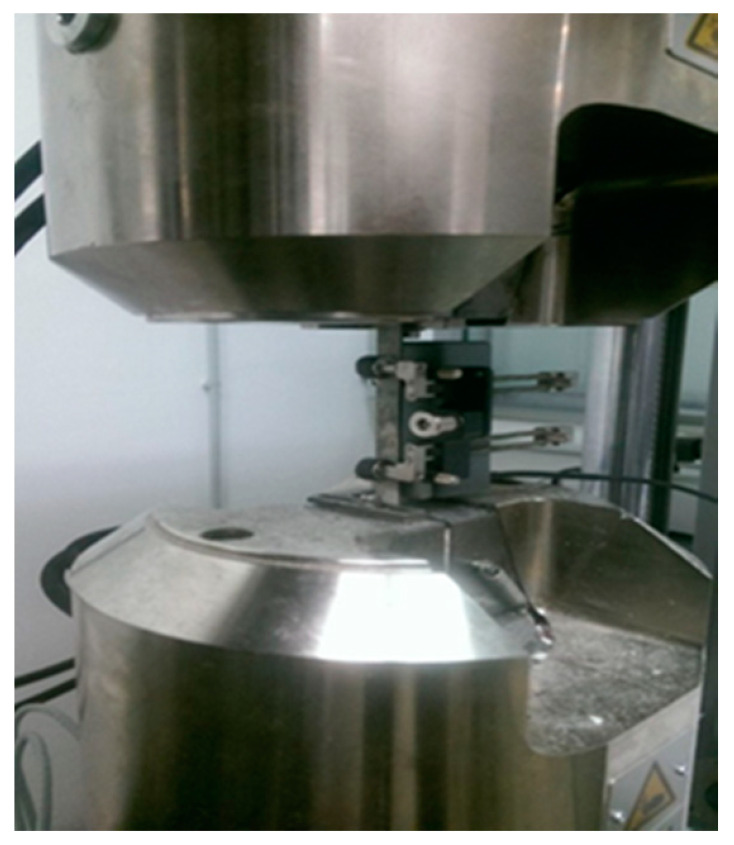
Tension specimen on the grips.

The tensile tests were carried out, at room temperature, with the testing machine Vibrophore 100, from Zwick/Roell Company (Ulm, Germany) with the software TestExpert III. In particular, the tensile strength of the BFRP composites was measured with specimens of dimensions 150 mm × 13 mm × 2.1 mm (Figure 3) with the gripping sections to be each 40 mm, giving a clear tension testing length of 70 mm (Figure 4). Furthermore, the crosshead speed was set at 1 mm/min. For this kind of experiment, five samples were tested, and the average values of them are presented in Table 3.

## 3. Results

### 3.1. DMA Tests

#### 3.1.1. Displacement Sweep Test

**Figure 5:**
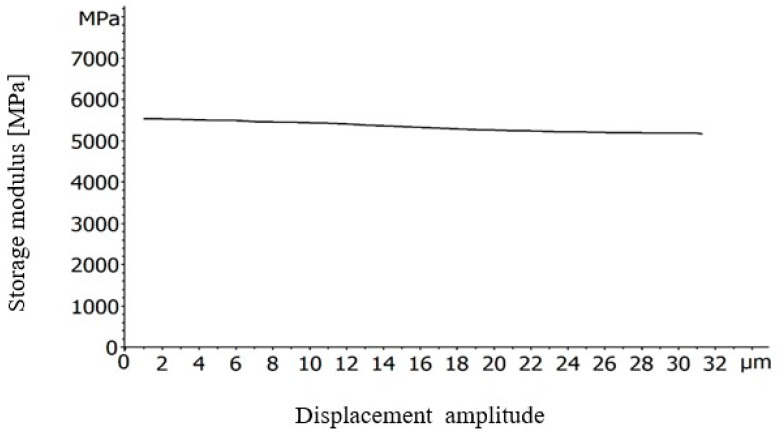
Displacement sweep test of the BFRP specimen; storage modulus versus displacement.

Figure 5 depicts the graph of a displacement sweep test on BFRP compound specimen. At the chosen 8 μm, the initial storage modulus value does not change remarkably so it is assumed that the DMA experiments take part in the linear viscoelastic region of the materials.

#### 3.1.2. DMA Experiments

**Figure 6:**
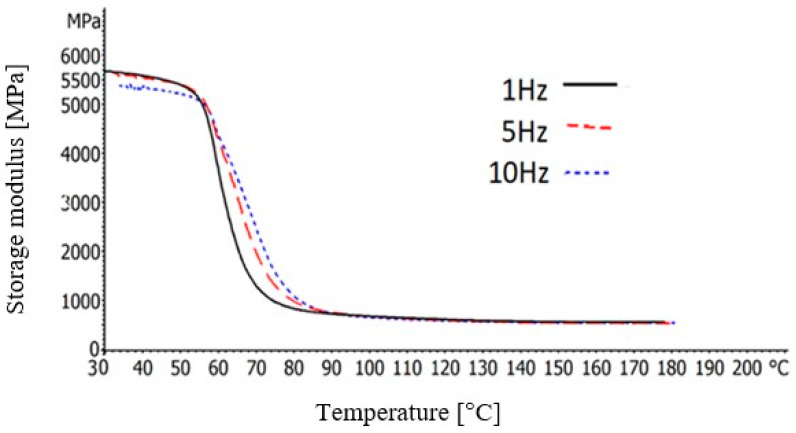
Storage modulus of the BFRP composites, as a function of the temperature, at 1, 5 and 10 Hz.

The storage modulus (E’) is associated with the stored energy of the materials. Figure 6 shows the storage modulus of the BFRP composites, as the temperature is increased, at three different frequencies; 1, 5 or 10 Hz. From this graph, it is observed that as the temperature is raised the storage modulus of the BFRP composites is reduced, with high rate, up to the 90 °C. Remarkably, after about the 55 °C, a steep drop in the values is observed. Generally, this steep fall in the storage modulus curve specifies the maximum working temperature of the materials and it is associated with the *T_g_*. However, it should be noted that as the frequency is increased, this transition region becomes less abrupt and with longer duration. Furthermore, after about the 90 °C, the storage modulus values are in a steady low value line until the final temperature.

**Figure 7:**
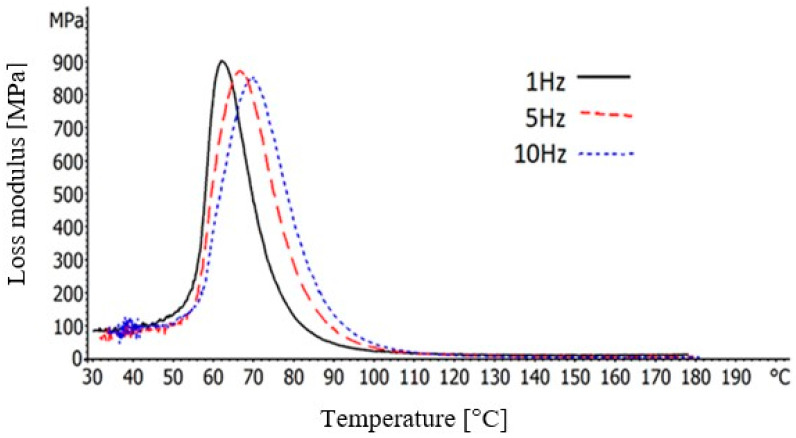
Loss modulus of the BFRP composites, as a function of the temperature, at 1, 5 and 10 Hz.

The loss modulus (E’’) is correlated with the lost energy, as form of heat, under DMA tests. High values of loss modulus point out viscous behavior therefore remarkable damping properties [25]. The loss moduli of the BFRP composites, at three different frequencies and as a function of temperature, is presented in Figure 7. As it can be seen, at low temperatures, the curves follow a steep increase achieving peak values whereas then a steep fall is observed until almost zero values which are maintained up to the final 180 °C. It is worth mentioning that higher frequency seems to have a negative impact in the peak values of the loss modulus.

The tanδ is the ratio of loss modulus to storage modulus

tanδ = E”/E’(1)

and it is sometimes called damping factor.

**Figure 8:**
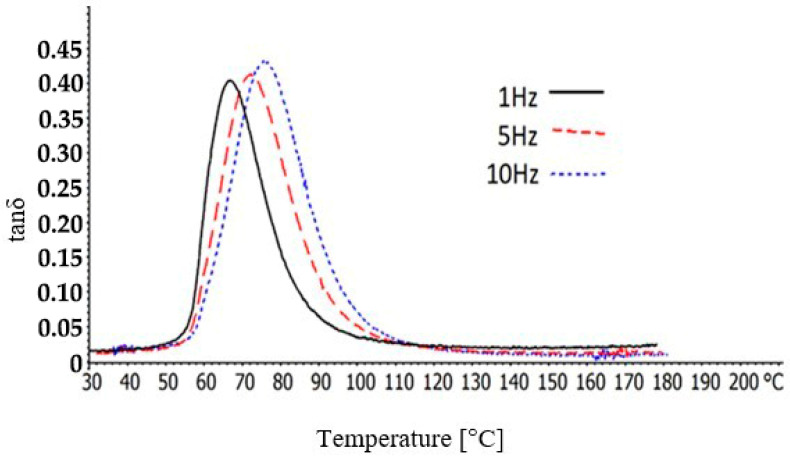
tanδ of the BFRP composites, as a function of the temperature, at 1, 5, and 10 Hz.

The tanδ curves of the BFRP composites, over the temperature range of 30–180 °C, whereas the frequency is 1, 5, or 10 Hz, are shown in Figure 8. As it can be seen, at low temperatures the values are almost stable, whereas after approximately the 50 °C, they follow a step upward trend, achieving a peak value, which can be used for the identification of the materials’ glass transition temperature, and then they follow a steep decrease.

Table 1 depicts the *T_g_* of the BFRP composites which were acquired through the corresponding temperature of the peak of the tanδ curves. It can be revealed that as the frequency is increased the BFRP can obtain higher *T_g_*. Daohai Zhang et al. [26] determined, through tanδ peak, the *T_g_* of long glass fiber reinforced thermoplastic polyurethane/poly(butylene terephthalate) composites, and they found the *T_g_* of these composites to shift in higher temperatures as the frequency was increased. Moreover, the *T_g_* of auto polymerized hard direct denture reline resins, determinate through dynamic mechanical analysis, was found to be higher in greater frequencies [27].

### 3.2. Thermomechanical Analysis (TMA)

#### 3.2.1. Creep Recovery Tests

**Figure 9:**
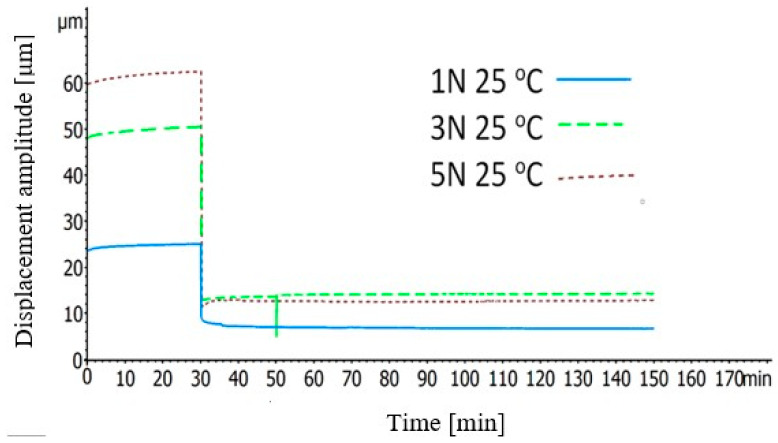
Creep recovery isothermal curves of the BFRP composites under various initial forces.

Figure 9 depicts the creep recovery behavior of the BFRP composites with an initial force of 1, 3 or 5 Newton at temperature 25 °C. In this graph, it can be noticed that the creep behavior of these composites is almost stable or very slightly increases during the first 30 min of the experiments whereas their recovery took part immediately after the release of the force. The final value of these curves is the permanent deformation which was caused on the composites and as it was expected the 1 Newton initial force caused the less.

**Figure 10:**
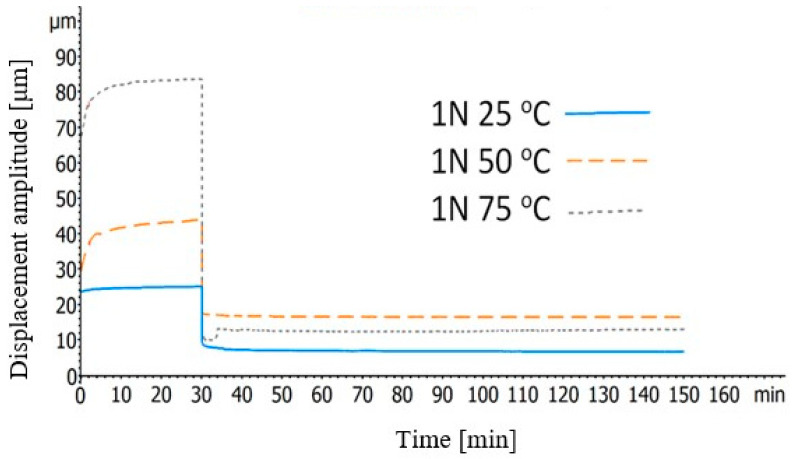
Creep recovery curves of the BFRP composites at various temperatures under initial force of 1 Newton.

Figure 10 illustrates the effect of the temperature on the creep recovery behavior of the BFRP composites. In this graph, a remarkable point is that, in higher temperatures, the resistance of the BFPR composites in deformation is reduced. Additionally, whereas at 25 °C, the deformation on the samples was almost steady during the first 15 min of the force application, at 50 and 75 °C, the deformation is rising rapidly during this time. This can be attributed to the fact that, at high temperatures, the structure of the composites becomes more compliant, thus the viscoelasticity becomes more evident and, as a consequence, this growing in deformation can be noticed.

#### 3.2.2. Stress-Relaxation Tests

**Figure 11:**
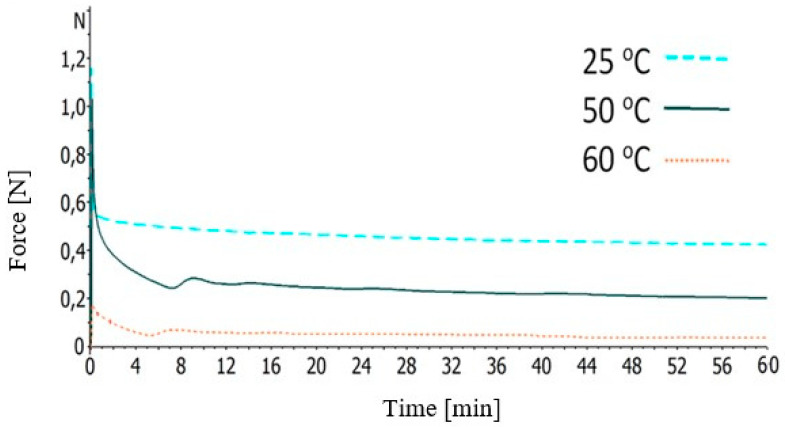
Stress-relaxation curves of the BFRP composites at various temperatures.

Figure 11 depicts the stress-relaxation of the BFRP composites at 25, 50, or 60 °C. Remarkably, the viscoelastic nature of the BFRP composites is visible in the first minutes of these curves; initially, the composites’ structure require a considerable high force which then is reduced, finally reaching almost stable values.

### 3.3. Flexural Experiments

**Figure 12:**
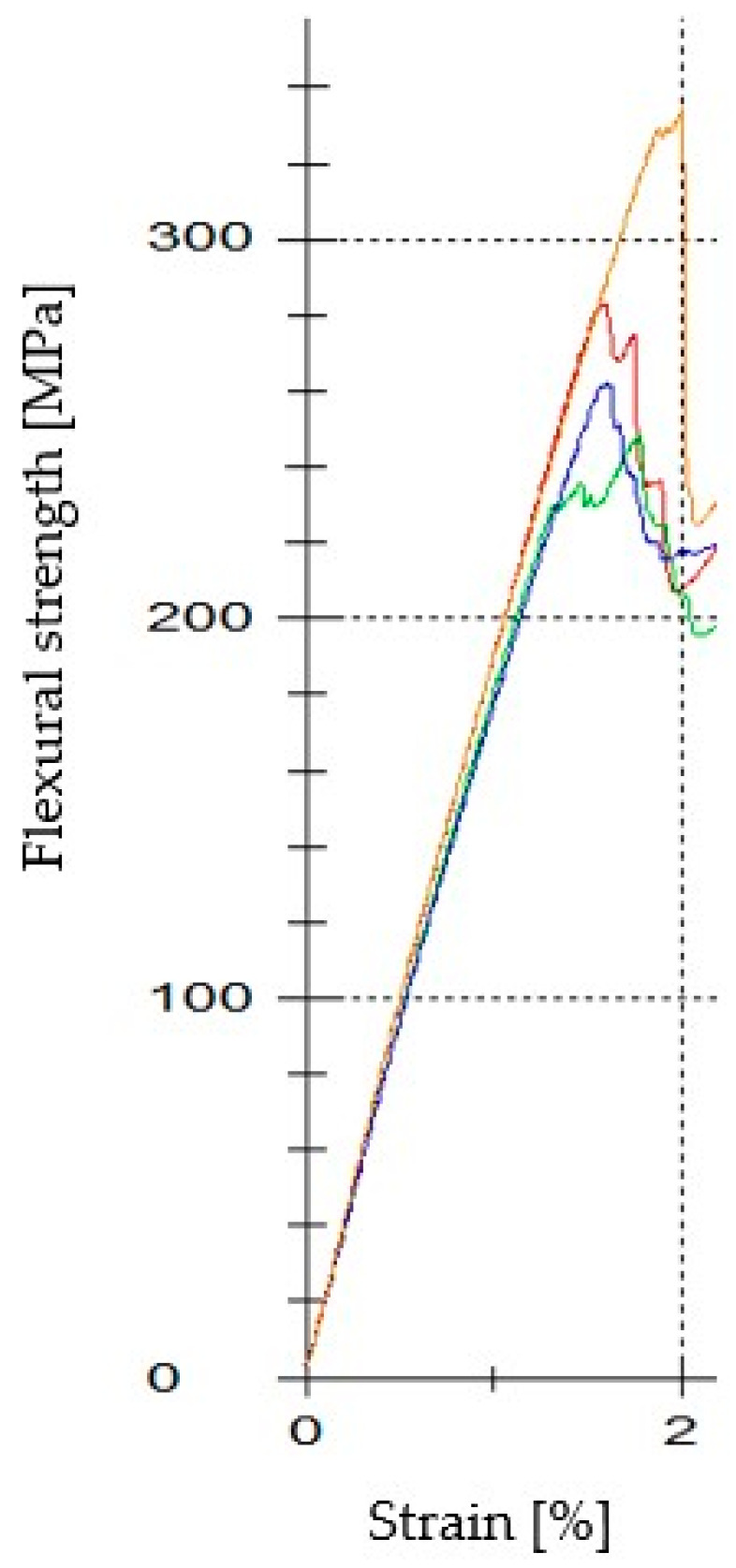
Flexural strength curves of the BFRP composites.

Strength of the materials is their ability to withstand forces without breakage. The determination of the flexural strength is very useful, especially in structural applications, whereas its identification also helps for the evaluation of the composites’ matrix–fiber interface. In engineering, the yield strength is the point at which the materials change from elastic to plastic deformation, and usually, it determines the maximum working strain of the materials, as after this point, there is permanent deformation on their structure. Table 2 shows the three-point bending experiments results of the BFRP composites, whereas Figure 12 represents the flexural curves of them in detail.

### 3.4. Tension Experiments

**Figure 13:**
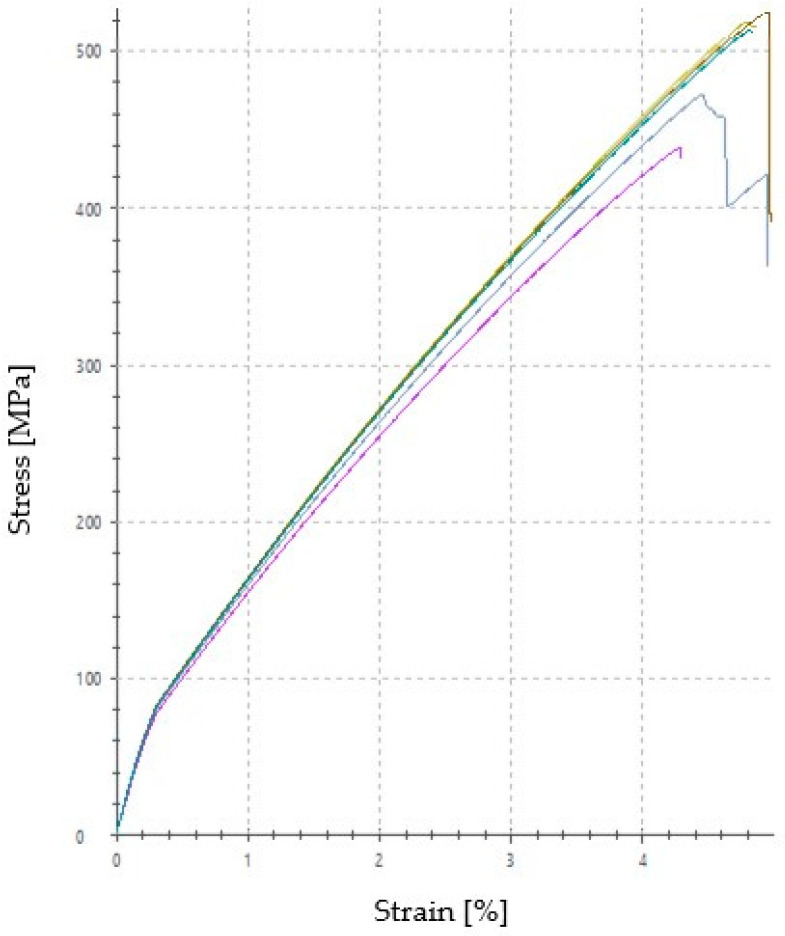
Tensile stress-strain curves of the five specimens.

The tension strength is the force which a material can withstand, due to pulling, before its breakage and it is one of the most important properties of the materials. The curves of the five tension experiments, are presented in Figure 13. As it can be revealed, a relative good reliability, for the hand lay-up fabrication method and composite heterogeneous structure, can be obtained, as three of them have almost identical curves, whereas the other two curves, despite the fact that they exhibited lower maximum values, follow the same general trend. Additionally, the BFRP composites are characterized as brittle materials because they break suddenly without showing plastic deformation.

### 3.5. TGA

**Figure 14:**
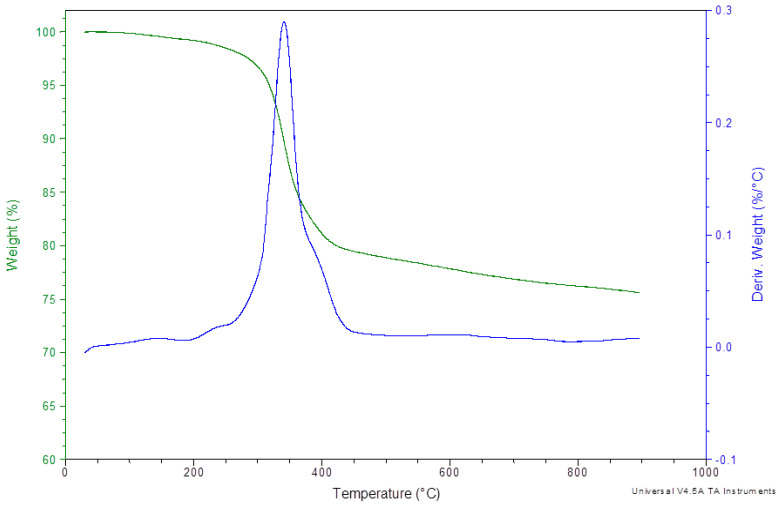
TGA and DTG curves of the BFRP composite in Nitrogen atmosphere.

**Figure 15:**
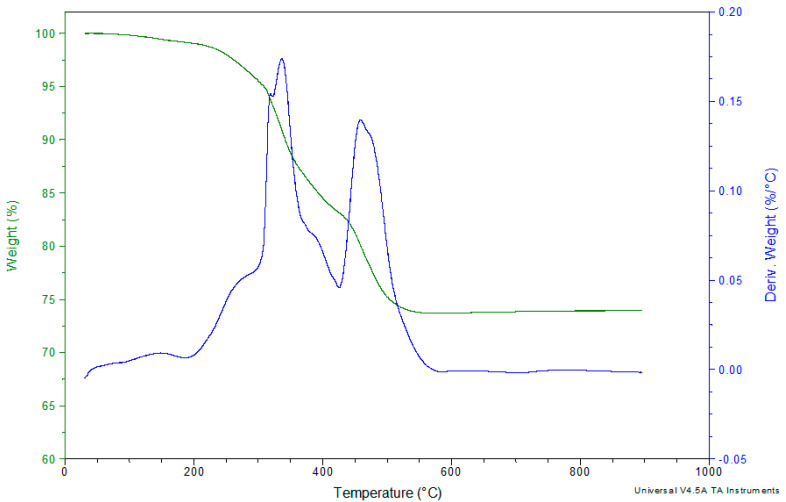
TGA and DTG curves of the BFRP composite in air atmosphere.

Figure 14 and Figure 15 show the weight of the BFRP composite as a function of the temperature, in the range 30–900 °C, in air or nitrogen atmosphere. As it can be seen, during the decomposition of the BFRP composite in both atmospheres, a sharp decline starts at approximately 260–300 °C, and this is attributed to the main weight loss of the epoxy matrix. Comparing the decomposition of the BRFP compound in air with its decomposition in nitrogen atmosphere, it can be revealed that the degradation of its matrix, as the basalt fibers are not thermally affected in this temperature, in the latter atmosphere takes part with a lower rate and continues up to the final 900 °C whereas in air the epoxy has completely decomposed up to the 550 °C. A remarkable point in the DTG graphs, is the maximum peaks which correspond to the maximum degradation rate of the investigated materials. In the case of the BFRP composite, TGA in N_2_, one peak is appeared at 341 °C, which is dedicated to degradation of its matrix, whereas in air, two maximum heating rates are presented at 336.4 and 458.6 °C, respectively.

**Figure 16:**
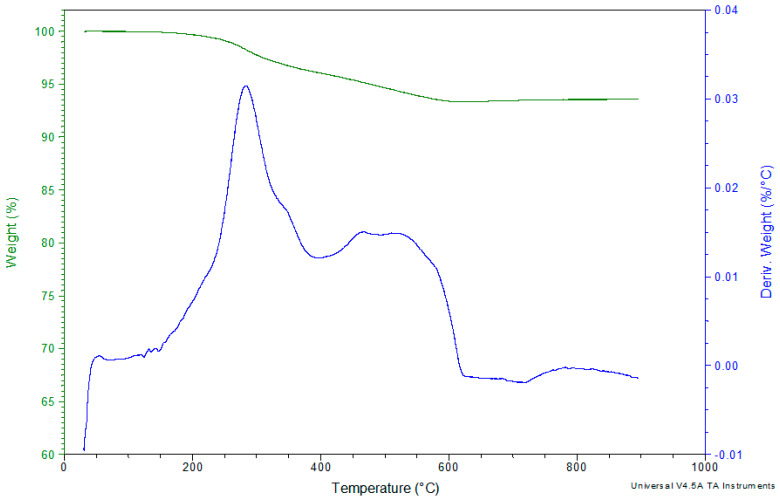
TGA and DTG curves of the basalt fibers in Nitrogen atmosphere.

**Figure 17:**
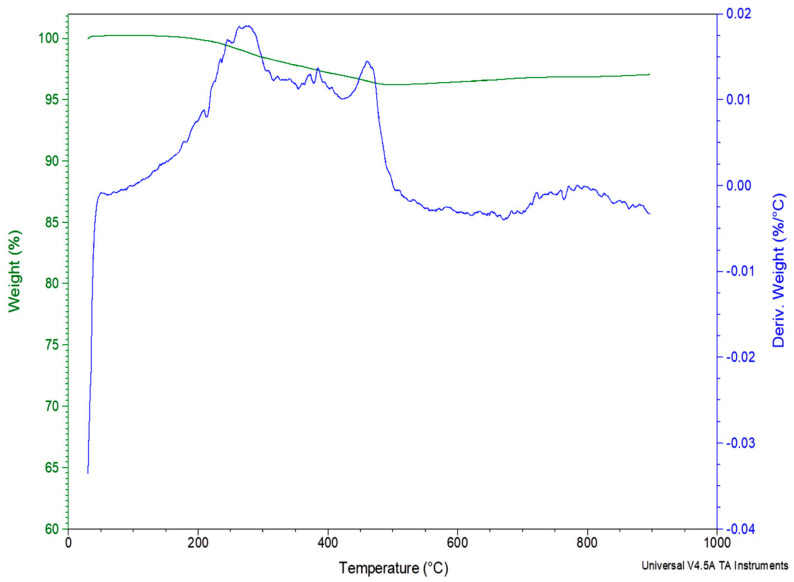
TGA and DTG curves of the basalt fibers in air atmosphere.

Figure 16 and Figure 17 depict the decomposition of the basalt fibers in air or N_2_ atmosphere. As it can be revealed, these fibers show excellent thermal resistance; they are not thermally influenced under both atmospheres up to the final 900 °C.

The used basalt fibers, in this TGA experiment, were taken from over the edges of the hand lay-up mold so they were not absolutely pure. The approximately 6% weight reduction, in the temperature range 200–600 °C, is attributed in decomposition, of a very small part of epoxy which was stuck on the basalt fibers during the composites’ production. This is also verified from the fact that after the approximately the 630 °C the basalt fibers are not thermally affected.

The used basalt fibers, in this TGA experiment, were taken from over the edges of the hand lay-up composite plate so they were not absolutely pure. The approximately 4% weight reduction, in the temperature range 200–500 °C is attributed in decomposition, of a very small part of epoxy which was stuck on the basalt fiber during the composites’ production. This is also verified from the fact that after approximately the 500 °C the basalt fibers are not thermally affected.

### 3.6. Calculation of the Fibers’ Volume Fraction (V_f_) and Volume of the Matrix (V_m_)

Based on the TGA experiments, and using the weight fraction of the reinforcement (*W_f_*), the weight fraction of the matrix (*W_m_*) and the densities of the basalt fibers and epoxy matrix, the *V_f_* of the reinforcement in the BFRP composite was calculated. In detail, the decomposition curve of the BFRP composite, in air atmosphere, after approximately the 550 °C became flat and steady, thus meaning that the epoxy matrix was completely decomposed where at the same time the basalt fibers were not thermally affected. At this temperature, the weight percentage is 73.76% and so this includes only basalt fibers; thus, *W_f_* = 73.76% and *W_m_* = 26.24 %.

The densities of the basalt fibers and epoxy matrix are known, 2.67 and 1.10 g/cm^3^ respectively. So, the volume fraction of the reinforcement phase, in terms of weight fraction, was found according to the equation [28]:

(2) $$V_{f}=\frac{W_{f}*\rho_{m}}{W_{f}*\rho_{m}+W_{m}*\rho_{f}}$$

where:

*V_f_* = volume of the reinforcement

*V_m_* = volume of the matrix

*ρ_m_* = density of matrix

*ρ_f_* = density of reinforcement

So, finally, *V_f_* = 53.66% and *V_m_* = 46.34%.

## 4. Conclusions

During this research study, BFRP composites with relatively high *V_f_* over 50% were successfully fabricated and were experimentally investigated through various thermal analysis and mechanical behavior tests. At this point, it must be noted that the basalt fibers, which have natural origin, when they are used in polymer matrix composites, especially with high *V_f_*, give them biodegradable characteristics, an advantage which nowadays is becoming more and more important due to environmental concerns. Based on the experimental results, the following conclusions can be drawn. Through the creep recovery and stress-relaxation tests, the significant impact of the high temperatures on the materials’ structure was revealed. Especially near and over the *T_g_*, the BFRP polymer showed remarkable reduction in resistance in deformation forces. Thus, it can be concluded that the *T_g_* is a critical point in the polymer matrix composites’ behavior. Through the DMA, it was revealed that as the frequency increases, the peak values of the tanδ of the BFRP composite move to higher temperatures, leading to a greater *T_g_*. The *T_g_* is affected by the frequency.

One of the significant revelations in this research is the thermal resistance of the basalt fibers. In particular, in the TGA runs, these fibers showed no decomposition, in the temperature range 30–900 °C, in nitrogen or air atmosphere. Due to this characteristic, the basalt fibers can be classified as potential material for usage in a very broad range of applications, as the resistance to high temperatures is always a critical factor. These could be as thermal insulations against fire dangers, and also in the surrounding regions of exhaust systems of airplanes, cars, and vehicles. Moreover, due to the basalt fibers’ low density, the combination of them in polymer matrix results in a composite with low weight, which turns to very important materials’ characteristics such as high strength-to-weight and high stiffness-to-weight. These features make the BFRP composites ideal for commercial aircraft applications where the BFRP composite’ low weight will turn in reduction in airplanes’ fuel consumption, whereas also in the case of fire, these fibers will show remarkable thermal resistance and will continue to provide structural support up to high temperatures.

From an overall point of view, the BFRP composites showed very good tensile and flexural strength, something which means that the epoxy matrix formed a very good interfacial bond with the basalt fibers. Generally, the FRP composites demonstrate low mechanical behavior in case of a poor bond between matrix and fibers. Moreover, based on the overall results, especially on those of the mechanical behavior of the BFRP composites, as in those in which a various number of specimens were used for reliability, and due to the fact that the composite plate has remarkably stable thickness (±0.1 mm) through its structure, the hybrid hand lay-up compression molding technique can be characterized as efficient and reliable for the production of FRP composites, with a high *V_f_* of fibers of over 50% as the produced BFRP composites.

## Figures and Tables

**Table 1:** Glass transition temperature of the BFRP composites.

Frequency	1 Hz	5 Hz	10 Hz
*T_g_* (peak of tanδ)	67.1 °C	72.1 °C	75.4 °C

**Table 2:** Average values of the three-point bending results.

	F at 0.2% Plastic Deformation [N]	Upper Yield Point [N]	σ_fsmax_ [MPa]
BFRP composite	187.75	195	282

**Table 3:** Average values of the tension experiments results.

	F at 0.2% Plastic Strain [N]	σ_tsmax_ [MPa]	Strain at Breakage [%]
BFRP composite	2580	494.4	4.68
